# Achieving Absolute Molar Lipid Concentrations: A Phospholipidomics Cross-Validation Study

**DOI:** 10.1021/acs.analchem.1c03743

**Published:** 2022-01-13

**Authors:** Harald Schoeny, Evelyn Rampler, Dinh Binh Chu, Anna Schoeberl, Luis Galvez, Markus Blaukopf, Paul Kosma, Gunda Koellensperger

**Affiliations:** † Department of Analytical Chemistry, Faculty of Chemistry, University of Vienna, Waehringer Str. 38, 1090 Vienna, Austria; ‡ Vienna Metabolomics Center (VIME), University of Vienna, Althanstraße 14, 1090 Vienna, Austria; § Chemistry Meets Microbiology, Althanstraße 14, 1090 Vienna, Austria; ∥ School of Chemical Engineering, Hanoi University of Science and Technology, 1 Dai Co Viet, Hai Ba Trung, Hanoi 100000, Vietnam; ⊥ Department of Chemistry, University of Natural Resources and Life Sciences Vienna, 1190 Vienna, Austria

## Abstract

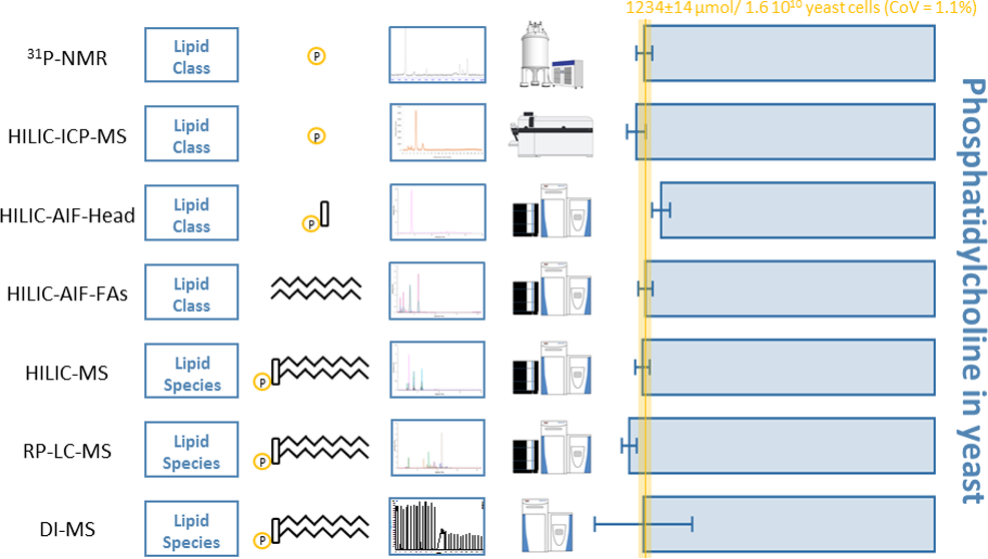

Standardization is essential in lipidomics and part of a huge community effort. However, with the still ongoing lack of reference materials, benchmarking quantification is hampered. Here, we propose traceable lipid class quantification as an important layer for the validation of quantitative lipidomics workflows. ^31^P nuclear magnetic resonance (NMR) and inductively coupled plasma (ICP)–mass spectrometry (MS) can use certified species-unspecific standards to validate shotgun or liquid chromatography (LC)-MS-based lipidomics approaches. We further introduce a novel lipid class quantification strategy based on lipid class separation and mass spectrometry using an all ion fragmentation (AIF) approach. Class-specific fragments, measured over a mass range typical for the lipid classes, are integrated to assess the lipid class concentration. The concept proved particularly interesting as low absolute limits of detection in the fmol range were achieved and LC-MS platforms are widely used in the field of lipidomics, while the accessibility of NMR and ICP-MS is limited. Using completely independent calibration strategies, the introduced validation scheme comprised the quantitative assessment of the complete phospholipid sub-ome, next to the individual lipid classes. *Komagataella phaffii* served as a prime example, showcasing mass balances and supporting the value of benchmarks for quantification at the lipid species level.

## Introduction

To date, accurate absolute quantification remains a grand challenge in lipidomics.^1^ Standardization is inherently difficult in omics type of analysis as the number of lipid species in a biological sample is high (typically several hundred) and the concentration ranges can cover several orders of magnitude (e.g., 8 in plasma^2^ or 7 in platelets^3^). We have seen huge progress in standardization,^2,4−6^ driven by valuable community efforts. However, the highest metrological order methods requiring traceable, certified reference materials are not yet routine. Certification is a complex process, which includes compositional and quantitative data as well as characterized stability and uncertainty for each analyte. Until 2021, this stage has not been reached in lipidomics.^7^ Diverse international ring trials were of paramount importance for harmonization in the field. In 2017, an interlaboratory comparison with more than 30 participants established consensus values for 339 lipids in the human plasma standard reference material (SRM) 1950 provided by the National Institute of Standards and Technology (NIST).^8^ An international ring trial has further delivered consensus values for 250 metabolites, including lipids, based on the Biocrates AbsoluteIDQ p400HR kit.^9^

In lipidomics, it is common practice to calibrate by class-specific internal standards (ISTDs) using a limited set of nonendogenous lipid species (e.g., short, long, or odd fatty acyl chains to avoid overlaps).^10^ Species-specific internal standardization by stable isotope-labeled analogues is the method of choice when aiming at accurate absolute quantification.^11^ However, comprehensive lipidome analysis by species-specific isotope dilution covering several hundreds of lipids is challenging. Despite progress in the availability of lipid standard panels^7^ and the use of isotopically labeled ISTDs,^12^ assay commutability (reproducibility of quantitative data obtained from different platforms) is still a bottleneck in lipid quantification.^11^ Normalization to SRM 1950 or to quality control (QC) samples was suggested to ameliorate harmonization and finally lead to assay commutability. As a drawback, no traceability is achieved by this strategy, as concentrations are traced back to consensus values that are not traceable themselves. This problem is highlighted in different seminal studies,^13−15^ which emphasize the challenge of integrating validation schemes and cross-platform comparisons.

In this work, we introduce a validation scheme based on traceable orthogonal quantification of lipid classes. Lipid class quantification has a long-standing tradition in the science of lipids;^16^ however, with the emergence of omics tools, this application became less important. Table 1 summarizes methods and their analytical figures of merit.

**Table 1:** Summary of Published Phospholipid (PL) Class Quantification Strategies^a^

**separation technique**	**analyzer/detector**	**LOD (nmol)**	**analyte**	**reference standard**	**paper**
	^31^P NMR	600	^31^P	P-containing ISTD	Kato^17^
	2D-^31^P,^1^H NMR	4	^31^P	P-containing ISTD	Kaffarnik^18^
	colorimetry	14–140	total PL	P-containing ESTD	Stewart^19^
	fluorometry	0.5	total PL	P-containing ESTD	Nanjee^20^
enzymatically	UV/vis	2.8	PA	class-specific ESTD	Dippe^21^
enzymatically	UV/vis	0.02	SM	class-specific ESTD	He^22^
TLC	autoradiography	0.9	^32^P	P-containing ESTD	Stephens^23^
TLC	densitometry	0.3	lipid class	class-specific ESTD	Weerheim^24^
TLC	colorimetry	0.2	P	P-containing ESTD	Zhou^25^
TLC	RP-LC-UV/vis	0.01	lipid class, derivatized	class-specific ESTD	Rastegar^26^
IC	conductivity	0.1	lipid class, deacyl. HG	class-specific ESTD	Nasuhoglu^27^
offline-IC	autoradiography	0.09	^32^P, deacyl. HG	P-containing ESTD	Stephens^23^
NP-LC	RI	1.0	universal	class-specific ESTD	Grit^28^
HILIC	ELSD	0.00014	nonvolatile comp.	class-specific ESTD	Giuffrida^29^
HILIC	CAD	0.007	nonvolatile comp.	class-specific ESTD	Kiełbowicz^30^
SFC	CAD	0.0009	nonvolatile comp.	class-specific ESTD	Takeda^31^
NP-LC	ICP-MS	0.007–0.04	P	P-containing ISTD	Kovačevič^32^
HILIC	ICP-MS	0.003–0.009	P	P-containing ISTD	Vosse^33^
HILIC	ESI-MS	0.07	lipid class, TIC + RF	retained ISTD	Cífková^34^
SFC	ESI-QTOF-MS	0.007	lipid class, TIC + RF	retained ISTD	Bartosova^35^
CE	ESI-IT-MS	0.0015	lipid class, deacyl. HG	class-specific ESTD	Warren^36^

a Limit of detection (LOD) values have been converted to the same unit (nmol) in absolute amount corresponding to LOD on column in chromatography. If only masses (e.g., ng) of lipid classes are given, an average molar mass of 700 g mol^–1^ was estimated. ESTD, external standards; TLC, thin-layer chromatography; IC, ion chromatography; SFC, supercritical fluid chromatography; CE, capillary electrophoresis; RI, refractive index; CAD, charged aerosol detector; QTOF, quadrupole time-of-flight; IT, ion trap; HG, head group; TIC, total ion chromatogram; RF, response factor; PL, phospholipid; PA, phosphatidic acid; SM, sphingomyelin.

We revisit class-specific quantification and explore its potential for mass balancing and thus benchmark current lipidomics workflows such as shotgun high-resolution mass spectrometry (HRMS), hydrophilic interaction liquid chromatography (HILIC)-HRMS, and reversed-phase liquid chromatography (RP-LC)-HRMS. While triglycerides and total cholesterol can be validated with clinical enzymatic tests,^37,38^ phospholipids (PLs) lack these widespread possibilities. Here, we address traceable PL class quantification in yeast by ^31^P nuclear magnetic resonance (NMR) and elemental mass spectrometry. Orthogonal methods allow the use of species-unspecific quantification by phosphorus, traceable to the Supporting Information (SI). While ^31^P NMR requires high sample amounts and long analysis time, which in turn demand stabilizing agents, inductively coupled plasma (ICP)-MS requires selective chromatographic separation of lipid classes. Recently, Vosse et al.^33^ discussed the analytical figures of merit of HILIC-ICP-MS analysis of PLs. Excellent absolute limits of detection <10 pmol were reported outperforming ^31^P NMR by at least 3 orders of magnitude.^17,18^ As a drawback, matrix dependence of ICP-MS analysis must be considered in species-unspecific quantification.

Additionally, we introduce LC-electrospray ionization (ESI)-MS strategies for lipid class quantification. Only a few reports on lipid class quantification by LC-ESI-MS exist.^34,35,39^ Cífková et al.^34^ have used the intensities of the total ion chromatogram (TIC) to generate lipid class peaks. To further reduce the number of necessary standards, only one sphingosyl phosphoethanolamine (ISTD) has been used together with a response factor approach to compensate for differences in ionization efficiency. Here, an all ion fragmentation (AIF) approach will be combined to a class-specific chromatographic separation and will be validated for lipid class quantification by ^31^P NMR and ICP-MS analysis. AIF is considered as a data-independent acquisition (DIA) method as all ions are fragmented intensity-independently in a certain mass range in contrast to data-dependent acquisition (DDA), which is limited to identification. In lipidomics, AIF is either used for the determination of the fatty acyl chain distribution of each class^40^ or dedicated software solutions are necessary to resolve the chimeric spectra of DIA for lipid species quantification.^41^ In this project, the disadvantage has been used as a benefit by integrating a class-specific fragment or a mass range typical for a lipid class over a certain retention time range.

First, we discuss the analytical figures of merit of our method portfolio, revealing caveats and capabilities of the orthogonal platforms relying on independent calibrations. Second, we showcase the potential of using lipid class quantification as a validation scheme, by showing how the enabled mass balances and benchmarks support the stringent validation of quantification at the lipid species level. We apply the validation scheme to the analysis of PLs in the yeast *Komagataella phaffii* (often referred to by its obsolete name *Pichia pastoris*), which is well known in biotechnological industries. Extensive knowledge on the phospholipidome of *K. phaffii*(42−45) was the ideal starting point for our validation study.

## Experimental Section

### Methods

Nine different quantification methods using different platforms were used (see Table 2) and described according to the use of separation and analyzer in detail in the Supporting Information.

**Table 2:** Summary of the Applied Quantification Methods^a^

**nr.**	**abbreviation**	**level**	**separation**	**analyzer**	**mode**	**quantification**	**quantified classes**
1	P NMR	class		NMR		ISTD	PC, PE, PI, PS
2	HILIC-ICP-MS-ext.cal.	class	HILIC	ICP-MS		ESTD	PC, (PE), PG
3	HILIC-ICP-MS-std.add.	class	HILIC	ICP-MS		std. add.	validated method 2
4	FI-ICP-MS	total		ICP-MS		ESTD	total P
5	HILIC-ESI-AIF-Head	class	HILIC	ESI-MS	AIF	ESTD	PC, (PE), PG, LPC
6	HILIC-ESI-AIF-FAs	class	HILIC	ESI-MS	AIF	ESTD	PC, (PE), PG, LPC
7	HILIC-ESI-MS1	species	HILIC	ESI-MS	MS1	ISTD	PC, PE, PG, LPC
8	shotgun	species		nESI-MS	MS1/DIA	ISTD	PC, PE, PI, PS, PG, LPC
9	RP-LC-MS	species	RP-LC	ESI-MS	MS1	ISTD	PC, PE, PI, PS, PG, LPC

a PC, phosphatidylcholine; PE, phosphatidylethanolamine; PI, phosphatidylinositol; PS, phosphatidylserine; PG, phosphatidylglycerol; LPC, lysophosphatidylcholine.

Briefly, ^31^P NMR analysis followed the protocol of Kato et al.^17^ The sample was prepared in a surfactant solution and mixed with phosphoserine as ISTD. The sample was pH-adjusted to 6.9 ± 0.04 and measured on an Avance III 600 MHz (Bruker, Billerica, MA). The peaks of the obtained spectra were integrated manually.

ICP-MS analysis was used for the total PL content (by flow injection (FI)) and lipid class (by HILIC separation) quantification with ESTD via a phosphorus tracer. An Agilent 1260 Infinity Bio-Inert HPLC system was coupled with an Agilent 8800 Triple Quadrupole ICP-MS (both Agilent Technologies, Santa Clara, CA). An iHILIC P column (2.1 mm × 150 mm, 5 μm, HILICON, Upsala, Sweden) was used to obtain class separation of polar lipids. SPLASH Lipidomix Mass Spec Standard (Avanti Polar Lipids, AL) was used for both external calibration and standard addition via HILIC-ICP-MS. FI experiments were conducted without a column and tributyl phosphate acted as the reference standard for external calibration. The obtained chromatograms were smoothed before integration and integrated manually using MassHunter 4.6 (Agilent Technologies).

HILIC-ESI-MS analysis was based on the same column but connected to a Vanquish Horizon HPLC coupled to a high-field Q Exactive HF quadrupole-Orbitrap mass spectrometer (both Thermo Fisher Scientific, Waltham, MA). Lipid class quantification was conducted with ESTDs in AIF mode and lipid species quantification with SPLASH Lipidomix Mass Spec Standard as ISTDs in MS1 mode. Skyline (version 20.2) was used for MS1 data processing and on the MS2 level for AIF fatty acyl chain or product ion head fragments. Neutral loss head fragments in the AIF files were integrated manually with Qual Browser Thermo Xcalibur (version 4.0.27.19, Thermo Fisher Scientific).

Shotgun analysis was conducted as previously described elsewhere^45^ on a high-field Q Exactive HF quadrupole-Orbitrap mass spectrometer (Thermo Fisher Scientific), connected with a robotic nanoflow ion source TriVersa NanoMate (Advion BioSciences, Ithaca, NY). SPLASH Lipidomix was added as ISTD, and data processing was performed with LipidXplorer 1.2.8.

A Vanquish Horizon HPLC (Thermo Fisher Scientific) with an Acquity HSS T3 (2.1 mm × 150 mm, 1.8 μm, Waters, Milford, MA) was coupled to a high-field Q Exactive HF quadrupole-Orbitrap mass spectrometer and used for RP-LC-HRMS. Skyline (version 20.2) was used for peak integration, and R/R Studio was used for final data processing.

### Figures of Merit

Figures of merit follow the EURACHEM guideline, The Fitness for Purpose of Analytical Methods, 2nd edition (2014).^46^ MS-based LOD and limit of quantification (LOQ) were determined by multiplying the standard deviation of replicate (*n* = 5) injections of a low concentrated reference standard with 3 and 10, respectively. Calculations were based on standard deviations as obtained from ISTD and ESTDs, for lipid species-level and lipid class-level quantification, respectively. In ^31^P NMR, LOD and LOQ correspond to signal-to-noise ratios (S/N) of 3 and 10, respectively. The calibration range for methods based on external calibration was inspected visually, and a coefficient of determination *R*^2^ > 0.9 was set as a minimum.

### Determination of Concentration Locations across Multiple Platforms

The determination of concentration locations for lipid classes across multiple platforms followed the CCQM Guidance note of the Bureau International des Poids et Mesures (BIPM).^47^ No extreme outlier values were expected, but the uncertainty might differ between the applied values. Hence, the recommended use of uncertainty-weighted mean *x̅*_u_ was chosen (see formula 1).
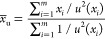
1

For each lipid class value, the inconsistency was checked via a chi-square test (see formula 2). Values above the critical χ^2^ value χ_0.05_^2^, *m –* 1 were considered as inconsistent, and the uncertainty (see formula 3) was corrected over observed dispersion (see Formula 4). Standard deviation *u*^2^ (*x*_*i*_) and *x*_*i*_ mean of technical replicates were used for calculation.
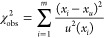
2
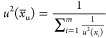
3
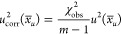
4

## Results and Discussion

### Lipid Class Quantification Methods

#### ^31^P NMR Analysis

**Figure 1:**
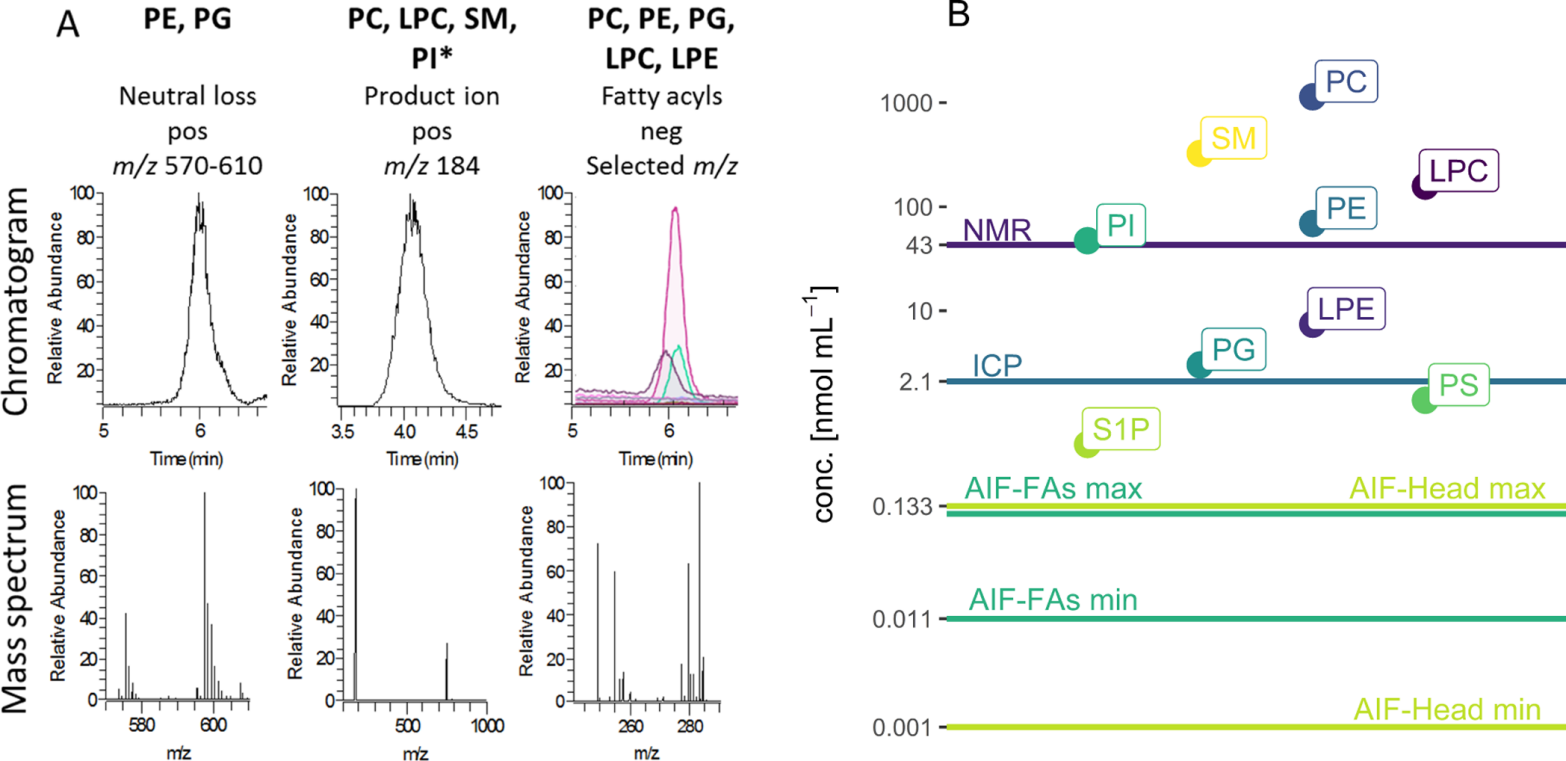
(A) Chromatograms and mass spectra of the three possible types in HILIC-ESI-AIF: Neutral loss, product ion (both head group fragments), and the sum of fatty acyl fragments. *PI functions after a similar approach but the head group fragment can be detected in negative mode at *m*/*z* 241. A detailed list can be found in Table S1. (B) Limits of detection in the solution of four lipid class quantification methods in comparison with lipid class concentrations in human plasma. Lines indicate LOD values for ^13^P NMR (purple, 42 nmol mL^–1^, 85 nmol in tube), FI-ICP (blue, 2 nmol mL^–1^, 21 pmol), maximal and minimal values for HILIC-AIF with the sum of fatty acids (FAs) (turquoise, 0.01–0.11 nmol mL^–1^, 55–560 fmol) the head group fragments (green, 0.001–0.133 nmol mL^–1^, 5–665 fmol). Exact values for all lipid classes can be found in Table S1. Lipid class conc. in human plasma represents the sum conc. for each class taken from the interlaboratory comparison of SRM 1950, NIST.^8^ LPE, lysophosphatidylethanolamine; S1P, sphingosine-1-phosphate.

^31^P NMR enables traceable species-unspecific standardization with the highest metrological order based on the phosphorus content of PLs.^17^ Exemplarily, Figure 1 in the Supporting Information gives a ^31^P NMR spectrum of the studied yeast *K. phaffii*. To control the pH of the measurement solution, the dried yeast extract was reconstituted in a surfactant-containing solution.^48^ A recovery of 92% was assessed using the standard PC 36:2 (0.5 μmol mL^–1^) for this reconstitution protocol, dedicated to ^31^P NMR analysis only. A certified standard of phosphoserine was used for internal standardization in ^31^P NMR. The limits of detection ranged at 43 nmol mL^–1^. As a consequence, the major abundant PL classes of PC, PE, PS, and PI could be absolutely quantified, while the low-abundant classes LPC and PG suffered from the reduced detection power (Figure 1B) and could only be assessed by mass spectrometry.

#### ICP-MS Analysis Using Flow Injection or HILIC Separation

ICP-MS constitutes an alternative approach for phosphorus quantification and thus traceable species-unspecific standardization of PLs.^32^ When omitting chromatographic separation, the entire sub-ome of the phospholipids can be absolutely quantified. The selectivity for PLs relies on a sample clean-up introduced by the tailored Folch extraction,^49^ selectively extracting lipids and removing otherwise abundant phosphorus-containing salts, macromolecules, and metabolites. In this work, high-throughput flow injection (FI) was implemented reducing the sample intake to a few microliters. A certified standard of tributyl phosphate served as an external calibrant. The LOD ranged at 2.1 nmol mL^–1^ (see Figure 1B) being superior to NMR by an order of magnitude. The method was linear over 2 orders of magnitude. When comparing the absolute quantities of the total phospholipidome in *K. phaffii*, excellent agreement was obtained with concentrations at 2340 ± 150 (6.4%) and 2250 ± 30 (1.3%) μmol/1.6 × 10^10^ cells for the entirely orthogonal ^31^P NMR (sum of PL peaks) and FI-ICP-MS, respectively. Finally, given the low sample input and sensitivity, the method proved to be a valuable harmonization tool. Recalibration of lipid species standards offered the assurance of standard stability ultimately to establish traceability (see Figure S3) in MS-based lipidomics.

To quantify individual PL classes, ICP-MS was combined with chromatography. HILIC is the established class separation method of polar lipids as retention is governed by the chemistry of the head group.^7^ HILIC is very versatile, but there is no single separation method, which is ideally suitable for the simultaneous measurement of acidic and neutral lipid classes. Therefore, compromised conditions are selected accepting severe peak tailing for acidic classes. As a result, high limits of detection, reduced column recoveries, and thus high uncertainties in quantification are observed for the classes of PI, PS, and phosphatidic acid (PA).^50^ When designing ICP-MS approaches, the selectivity of class separation is a must, as the quantification is based on phosphorus measurements only (see Figure S2). Gradient and matrix dependencies increase the uncertainty and have to be considered involving correction strategies by response factors^32^ or the implementation of isocratic conditions using counter gradient systems.^33^ In this work, the problem was overcome by lipid class-specific external calibration. Standard addition proved the calibration strategy fit for purpose. As a quality control measure, FI-ICP-MS runs of standards and samples preceded the actual HILIC-ICP-MS measurements.

#### HILIC-ESI-MS-Based All Ion Fragmentation (AIF) Analysis

The combination of HILIC and the ESI-MS-based AIF was only used for the determination of fatty acyl chain compositions so far.^40^ Here, we introduce HILIC-ESI-AIF as a novel strategy for lipid class quantification, relying on class-specific fragments and defined retention time ranges. Figure 1A exemplarily shows the selection of different fatty acyl chain fragments and the use of the neutral loss for PE and the product ion head group fragment for PC, while fragments for further classes can be found in Table S1. Thus, one peak with a defined mass and retention time window represents one lipid class either as a head group or as a fatty acyl chain fragment (Figures S4 and S5). In general, triple quadrupole instruments are as applicable as high-resolution instruments as neutral loss scans or product ion scans are common modes in tandem mass spectrometry. It is a clear limitation for MS2-based quantification strategies that the fragmentation efficiency correlates to fatty acyl composition (especially concerning the number of double bonds).^51^ Hence, the fatty acyl composition difference between standard and the lipid species needs to be considered. In the case of the investigated *K. phaffii*, the rather simple fatty acid profile facilitates the AIF strategy as the fatty acids FA 16:0, FA 18:1, FA 18:2, and FA 18:3 make up to 90% of the total fatty acid content in the cell homogenate. Only, minor contributions were found for FA 14:0, FA 16:1, FA 18:0, and FA 26:0.^44^ The HILIC-ESI-AIF concept is particularly interesting as low absolute limits of detection in the fmol range can be achieved (see Figure 1B), and LC-MS platforms are widely used in the field of lipidomics, while the accessibility of NMR and ICP-MS is limited at the same time having compromised sensitivity.

### Benchmarking the Lipid Class Concentration of *K. phaffii*

**Figure 2:**
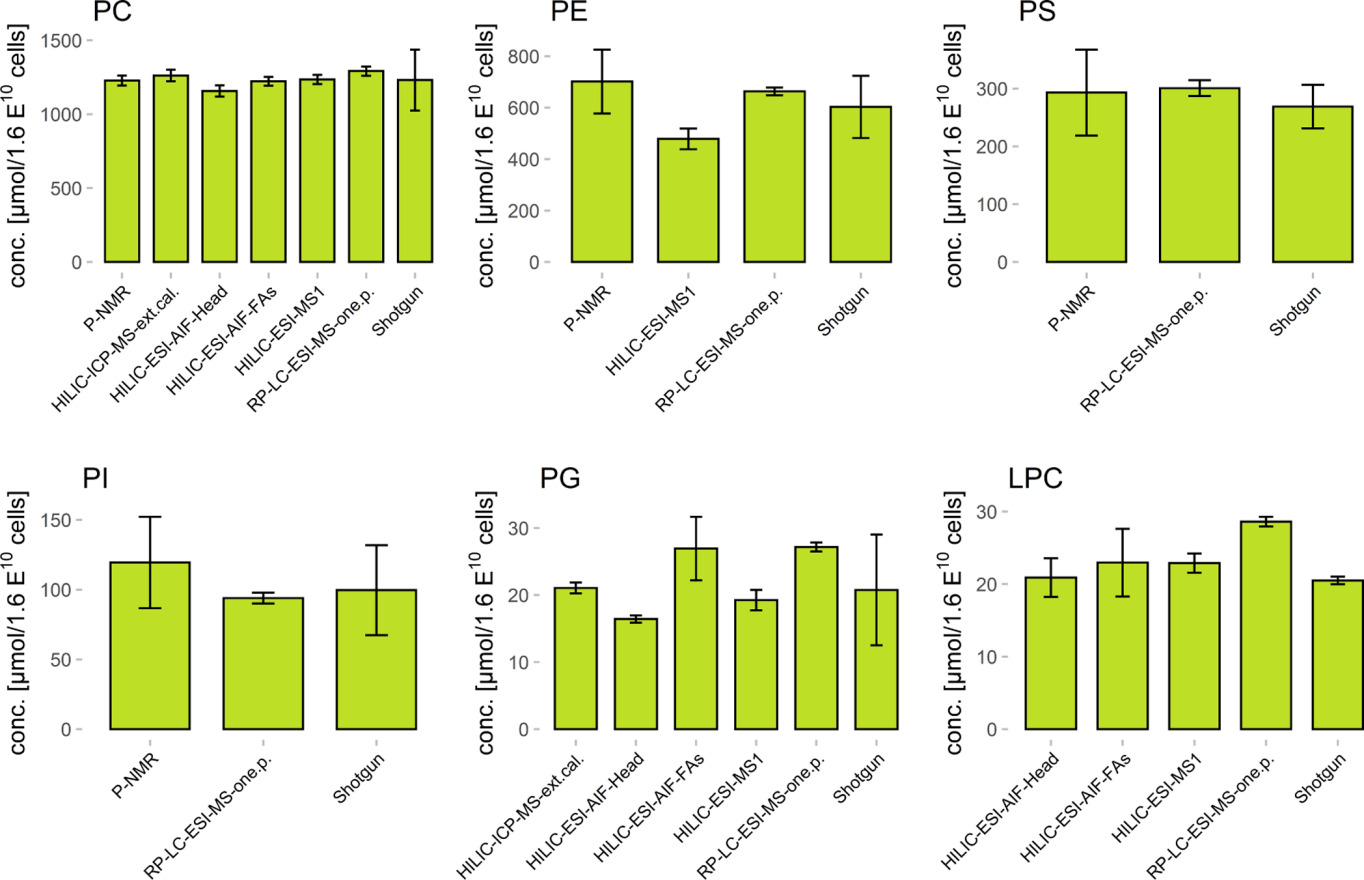
Absolute concentration values of different lipid classes in μmol/1.6 × 10^10^ yeast cells (equals 1 g wet weight of yeast cells). Error bars indicate technical repeatability (*n* = 3). Values can be found in Table S2. Classes with less than 7 bars were limited by sensitivity and selectivity. In detail, PE overlap with PA in HILIC methods, but as PA is relatively low concentrated, values still can be obtained as shown in Figure S6. Acidic lipids (PS, PI) were limited by their peak shape in HILIC separations reducing the sensitivity, while PG and LPC methods are only limited by the relatively low abundance in yeast.

The different class-specific strategies, namely, ^31^P NMR, HILIC-ICP-MS, and HILIC-ESI-AIF, were cross-validated calculating the uncertainty-weighted mean^47^ of the PL classes of the yeast *K. phaffii*. The implemented HILIC separation was not optimized for acidic lipid classes. Consequently, HILIC analysis of PA, PI, and PS was compromised showing a biased lower total PL concentration across all HILIC methods (1890 ± 35 μmol/1.6 × 10^10^ cells), which amounts to roughly 80% as assessed by ^31^P NMR and FI-ICP-MS. Table S2 summarizes the obtained lipid class methods results, while Figure 2 also benchmarks the data with lipid species methods. PC is the most abundant PL class followed by PE. For PC, all methods were above LOQ and could be included showing excellent agreement. Across all lipid class platforms, values for PC were consistent with a coefficient of variation (CoV) of 1.3% and a mean concentration of 1218 ± 17 μmol/1.6 × 10^10^ cells. The implemented HILIC method failed to selectively separate PE from PA; therefore, HILIC-based lipid class methods for PE are not shown in Figure 2. However, the amount of PA present in *K. phaffii* is rather low compared to PE (one study reported 2% PA vs. 31% PE of total PL content^44^); therefore, it was neglected, which overall results in a good agreement between the platforms with a mean value of 594 ± 8 μmol/1.6 × 10^10^ cells and a CoV of 1.3% (Figure S6). HILIC approaches were excluded for the acidic lipid classes PA, PI, and PS as the classes were found <LOQ. However, the latter classes could be quantified with ^31^P NMR as column saturation is not an issue and further preconcentration was possible as enough sample material was available. For the low-abundant class of PG, only HILIC-ICP-MS enabled traceable lipid class quantification. A mean concentration of 18 ± 1.6 μmol/1.6 × 10^10^ cells and a CoV of 9.4% were achieved. For the lipid classes of PG, PE, and PC, the novel HILIC-ESI-AIF-MS was validated successfully via ^31^P NMR and HILIC-ICP-MS. Therefore, for low-abundant LPC (<LOQ for ^31^P NMR/ICP-MS assessment), the two HILIC-ESI-AIF values were accepted as a reference point for mass balancing lipid species quantification. In summary, the appropriate method for lipid class quantification depends on the sample type, the available sample amount, the analytes of interest, and their lipid concentration. A general rating according to the metrological order of the methods is as follows: (1) ^31^P NMR, (2) HILIC-ICP-MS, (3) HILIC-ESI-AIF with head group fragments, and (4) HILIC-ESI-AIF with FA fragments.

### Mass Balancing Lipid Species Methods

**Figure 3:**
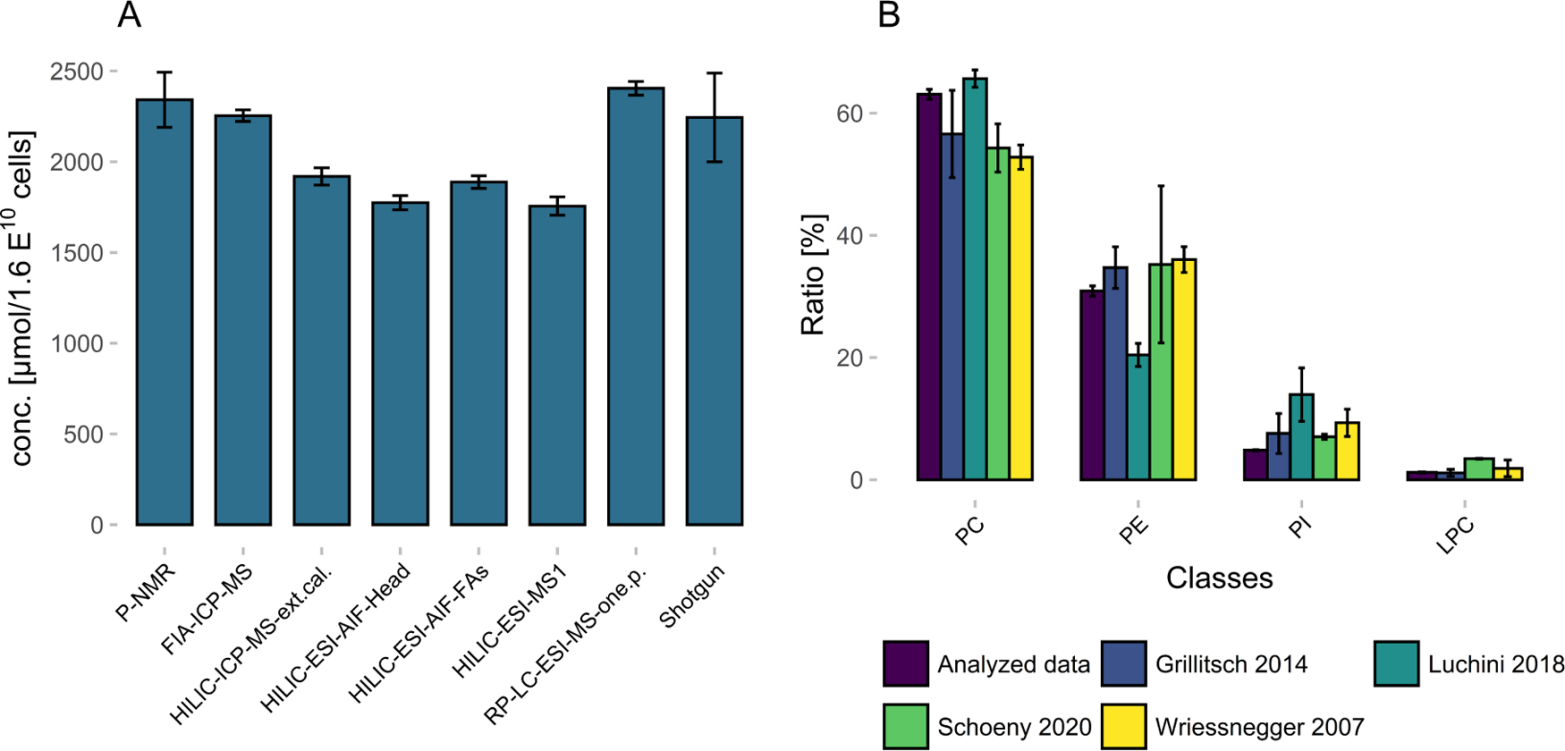
(A) Absolute concentration values for the total PL conc. in μmol/1.6 × 10^10^ yeast cells (equals 1 g wet weight of yeast cells). An average value of 1920 ± 60 μmol/1.6 × 10^10^ cells (CoV of 3.2%) for total PL conc. was achieved. (B) Data comparison with literature values. Analyzed data represent the mean value of the present study. Data in Grillitsch,^44^ Luchini,^43^ and Wriessnegger^42^ have been obtained by quantitative TLC and data from Schoeny^45^ by prep-SFC fractionation with a subsequent shotgun analysis. As some literature values are only shown in ratio values, the presentation in absolute concentration units is not possible. Only classes quantified by all studies are shown.

The limitations and critical points to consider in lipid species quantification are comprehensively described elsewhere.^10,52−54^ It is well accepted that RP-LC using only one ISTD per class is not the method of choice for accurate absolute quantification. This holds true especially when highly unsaturated lipids^50^ are analyzed. However, in the case of yeast lipidomics, this aspect is less important due to the lower complexity and lower degree of unsaturated fatty acids present compared to the well-investigated matrix of human plasma.^8,55^ Overall, co-ionization, achieved by minimal retention time differences between analytes and ISTD, is the key for accurate quantification.^10^ HILIC-MS and shotgun lipidomics, both approaches ensuring co-ionization, in turn, are compromised by isomeric overlaps, e.g., the classes PC and PE. In this work, the problem was solved as shotgun quantification was based on negative mode measurements of the fatty acyl chain fragments, and in HILIC-MS, selectivity was provided by chromatographic separation. Mass balances between the different methods showed overall good agreement. Excellent CoVs calculating the uncertainty-weighted mean^47^ were calculated considering all methods delivering lipid class quantities and the sum of all lipids quantified on the species level (see Figure 2 and Table S3). In total, a mean total concentration of 1920 ± 60 μmol/1.6 × 10^10^ cells with a CoV of 3.2% across all methods was achieved (see Figure 3B). It must be kept in mind that the observation cannot be generalized, as the investigated lipids are highly abundant, and the lipid species profile of yeast is not too complex. Correlation plots between the lipid species quantification methods (shotgun, HILIC, and RP-LC) are shown in Figure S7. A higher correlation of higher values in HILIC vs RP was found compared to low-concentration values. In shotgun analysis, concentrations <1 μmol/1.6 × 10^10^ cells fall below the limit of quantification. The obtained correlation graphs are in accordance with other studies^11,50^ as the majority of the lipids correlated to a high degree. At the same time, some lipid species exhibited differences of up to 1 order of magnitude. Lange et al.^50^ could correlate these offsets with the number of double bonds especially in the lipid classes PC and PE, which was confirmed in this study. Finally, the relative lipid class distribution across all quantified PLs enabled benchmarking with published data for *K. phaffii* (see Figure 3B). Hydrolyzed FAs and PL have been studied by gas chromatography (GC)-MS and TLC, respectively,^42−44^ and only our previous publication^45^ applied shotgun lipidomics as lipid species method after a pre-fractionation via prep-SFC. In summary, the data revealed a consistent picture. PC values range around 55%, followed by PE with approximately 35%. The relatively high LOD of TLC (see Table 1), the high standard deviation, and the varying values for PI and LPC between the published TLC methods also highlight the limitation of this time-consuming lipid class quantification method.

The better characterization of the *K. phaffii* yeast lipidome can help to establish a benchmarking tool for the development of an MS-based lipidomics method.^56,57^ The applied traceable lipid class quantification methods in combination with the simple and reproducible production of *K. phaffii* fermentation enable a cost-effective and accessible material, which is suitable as a QC system for long-term as well as large-scale studies. The proposed workflows pave the way for quantitative lipidomics studies including (1) species-unspecific standardization even for new PL classes by ^31^P NMR and ICP-MS and (2) support the development of lipid reference materials.

## Conclusions

In conclusion, values from nine different methods on the lipid species, lipid class, or total PL content level were obtained. ^31^P NMR—as a fully traceable method—can be recommended for the quantitative assessment of unknown samples or sample pools in bigger cohort studies. However, the high sample need and the long acquisition time make it impractical for direct sample comparison in larger cohort studies. Also, overlaps of lipid signals are still possible and need further improvement. ICP-MS is a useful alternative where species-unspecific quantification is also possible. Special care must be taken about the selected HILIC method to avoid overlaps and improve peak shape and lipid class recovery. The use of FI-ICP-MS is a simple method for the traceable quantification of less complex samples and shows great potential for the degradation assessment of phosphorus-containing standards. We further introduced a novel lipid class quantification based on HILIC and AIF. Depending on the applied HILIC conditions, this was valuable for the lipid classes of PC, PE, PG, and LPC. If available, universal lipid detectors, e.g., CAD and ELSD, are interesting alternatives with competitive LODs. Whatever lipid class quantification method is finally chosen, the benefit of cross-validated lipid concentrations was highlighted and can help to bring lipidomics further to a standardized and harmonized field.

## Supporting Information Available

Extended experimental section and supporting tables and figures (PDF)
