# Supplementary material for: Achieving Absolute Molar Lipid Concentrations: A Phospholipidomics Cross-Validation Study
Source: Anal Chem. 2022 Jan 13;94(3):1618–25. doi: 10.1021/acs.analchem.1c03743 (PMC8792901; doi:10.1021/acs.analchem.1c03743)
Supplement: Supplementary file 1 — ac1c03743_si_001.pdf [file ac1c03743_si_001.pdf]

Hunting absolute molar lipid concentrations: A phospholipidomics cross-validation study

Harald Schoeny[a], Evelyn Rampler[a,b,c], Dinh Binh Chu[a,d], Anna Schoeberl[a], Luis Galvez[a], Markus Blaukopf[e], Paul Kosma[e] and Gunda Koellensperger[a,b,c *]

[a] Department of Analytical Chemistry, Faculty of Chemistry, University of Vienna, Waehringer Str. 38, 1090 Vienna, Austria

[b] Vienna Metabolomics Center (VIME), University of Vienna, Althanstraße 14, 1090 Vienna, Austria

[c] Chemistry Meets Microbiology, Althanstraße 14, 1090 Vienna, Austria

[d] School of Chemical Engineering, Hanoi University of Science and Technology, 1 Dai Co Viet, Hai Ba Trung, Hanoi 100000, Vietnam

[e] Department of Chemistry, University of Natural Resources and Life Sciences Vienna, 1190 Vienna, Austria

* Corresponding author: E-Mail: gunda.koellensperger@univie.ac.at

Table of Contents

Experimental Section 2

Sample and materials 2

Methods 2

^{31}P-NMR analysis 2

ICP-MS analysis 3

HILIC-ESI-MS analysis 4

Shotgun analysis 5

RP-LC-MS analysis 6

Bibliography 7

Tables 8

Figures 9

EXPERIMENTAL SECTION

Sample and materials

The yeast sample extraction was conducted as described in Neubauer *et al.*[1] and Schoeny *et al.*[2] A final lipid extract from 16 billion cells (~10 mg lipid dry weight) was obtained and, if not other stated, dissolved in 1 mL chloroform and diluted 1:10, 1:100 and 1:1000 with the starting conditions. PC 36:2 was used for recovery experiments and was obtained from Avanti Polar Lipids, Inc. (Alabaster, AL, USA). For ^{13}P-NMR analysis, sodium cholate hydrate (99%, purchased from Sigma-Aldrich, Vienna, Austria), EDTA (99.995%, trace metals basis, Sigma-Aldrich), deuterium oxide (D_2O, 99.9%, Sigma-Aldrich), trizma® Base (99.7%, Sigma-Aldrich) and NMR test tubes with a 5-mm outer diameter (Sigma-Aldrich) were used. O-phospho-L-serine (CRM, TraceCert®, Sigma-Aldrich) was used as ISTD. For chromatographic methods, acetonitrile (ACN) and water were of LC-MS grade and ordered at Honeywell (Vienna, Austria). Chloroform, isopropanol (IPA), and methanol (MeOH) were also of LC-MS grade and ordered at Fisher Scientific (Vienna, Austria). Ammonium acetate and ammonium formate were ordered as eluent additives for LC-MS at Sigma Aldrich. SPLASH® Lipidomix® Mass Spec Standards were purchased from Avanti Polar Lipids, Inc. Tributyl phosphate (CRM, Supelco®, Sigma-Aldrich) was used as ESTD in FI-ICP-MS.

Methods

Nine different quantification methods using different platforms were used (see Table 1 in the manuscript) and are described in the following sections according to the applied separation technique and the analyzer. The different modes and quantification strategies using the same separation technique and analyzer are combined in the same section.

^{31}P-NMR analysis (lipid class quantification with ISTD)

The protocol followed Kato *et al.* 2018.[3] Following solutions were prepared: surfactant solution (20%, 1 g sodium cholate in 5 mL D_2O), Tris HCl Buffer (1 M, pH 7), EDTA solution (100 mg mL^{-1} in Tris HCl Buffer), an ISTD solution (3.8 mM, 1 mg mL^{-1}, 20 mg phosphoserine in 2 mL EDTA solution and 18 mL Tris HCl Buffer) and a PC 36:2 standard solution (1 mM in chloroform). The dried lipid extract from 16 billion cells and 1 mL of the PC 36:2 standard solution, which was dried down before, were dissolved in a 1 mL surfactant solution. To each of them, 1 mL ISTD solution was added and shaken for 1 h at 50°C before it was centrifuged at 15.000 rpm. The obtained supernatant was pH adjusted to 6.9±0.04 using 1 M NaOH or 1 M HCl solutions. Following conditions were used for ^{31}P-NMR measurements on the Avance III 600 MHz (Bruker, Billerica, MA, USA): irradiation frequency (242 MHz), acquisition time (5.4 s), probe temperature (30°C), spectral width (80 ppm), FID data points (210934), spinning (15 Hz), dummy scans (4), 1H decoupling (Waltz16), pulse angle (90°) pulse width (14.8 µs). The

peaks of the obtained spectra were integrated manually, the integral of the ISTD was normalized to 1, and the final lipid class concentration was calculated as in formula 1:

$$(1) \qquad conc\left[\frac{\mu mol}{1\ mL \approx 1.6\ 10^{10}\ cells}\right] = Norm.integral_{\ Analyte} \cdot conc\left[{}^{\mu mol}/_{mL}\right] \cdot Dil.factor$$

Limit of detection (LOD) was calculated as the conc. that corresponds to a signal-to-noise ratio (S/N) of 3. The noise value was obtained by integrating a region without peaks with peak width similar to analyte peaks in the spectrum (5.793-5.291 ppm).

ICP-MS analysis (total phospholipid content with FI and lipid class quantification with HILIC)

For HILIC-ICP-MS measurements an Agilent 1260 Infinity Bio-Inert HPLC system was coupled with an Agilent 8800 Triple Quadrupole ICP-MS (both Agilent Technologies, Santa Clara, CA, USA). An iHILIC® P column (2.1 mm x 150 mm, 5 µm, HILICON, Upsala, Sweden) connected to a PEEK -inlet filter was used to obtain class separation of polar lipids. A mixture of ACN/H_2O (50:50, v/v) with 10 mM ammonium acetate at pH 8.0 was used as eluent A and a mixture of ACN/H_2O (95:5, v/v) with 10 mM ammonium acetate at pH 8.0 was used as eluent B. The applied gradient was as follows: 0-2 min 100% B, 2-12 min gradient to 80% B, hold from 12-16 min, at 16 min switched back to 100%, and hold for column equilibration from 16-22 min. The flow rate was kept constant at 250 µL min^{-1} and the column oven at 40°C. The injector needle was washed with 75% IPA, 24.9% H_2O, 0.1% formic acid before each injection. SPLASH® Lipidomix® Mass Spec Standard (Avanti Polar Lipids) was used for both external calibration and standard addition via HILIC-ICP-MS. FI (Flow injection) experiments have been applied without the column, an injection volume of 10 µL, a flow of 250 mL min$^{-1,}$ and a constant eluent mixture of 50% A/ 50% B. Tributyl phosphate was used as the reference standard for external calibration via FI. The sample introduction system consisted of a MicroMist™ nebulizer (AHF Analysentechnik AG, Tuebingen, Germany) and a quartz double-pass spray chamber (Agilent Technologies). The instrument was tuned on a daily basis in order to achieve maximum sensitivity while keeping the oxide formation ($^{140}Ce^{16}O^+/^{140}Ce^+$) and the doubly charged ratio ($^{140}Ce^{2+}/^{140}Ce^+$) below 2%. Phospholipids were detected via phosphorus in the oxygen gas mode using mass shift modality ($^{31}P^+ \rightarrow {}^{31}P^{16}O^+$). Identification of phospholipid in the samples was performed via retention time on the LC column and the phosphorus tracer in ICP-MS.

For the ICP setting the following optimized conditions were used: Plasma gas 15 L min^{-1}, auxiliary gas 0.8 L min^{-1}, carrier gas 0.80 L min^{-1}, optional gas flow rate 35% (of carrier gas flow rate), rf power 1550 W (reflected power 10 W), ORC gas (oxygen) 30%, spray chamber temperature 2°C and sample depth (torch-interface distance) 7.8 mm. All chromatograms were smoothed before integration by using MassHunter software (Version 4.6, Agilent Technologies).

Results from external calibration were obtained via linear regression models of ESTDs and multiplying the results with the applied dilution factor (1:10 and 1:100). LOD was calculated by multiplying the standard deviation of 5 repetitive injections of a low concentrated recovery standard with 3. The external calibration for FI and HILIC-ICP-MS respectively was used to calculate the LOD concentration. Additionally, standard addition was applied for validation, and concentration values were obtained by multiplying the x-intercept value of a linear regression model (sample and sample + SPLASH® at different conc. levels) with the applied dilution factor (1:100).

HILIC-ESI-MS analysis (lipid class quantification with ESTD in AIF mode and lipid species quantification with ISTD in MS1 mode)

For the organic MS method, the same HILIC method (column, eluents, gradient, temperature, and flowrate) was applied as in the HILIC-ICP-MS analysis. However, a Vanquish™ Horizon HPLC was used instead and coupled to a high field Q Exactive HF™ quadrupole-Orbitrap mass spectrometer (both Thermo Fisher Scientific, Waltham, MA, USA).

With the same chromatographic method, two modes can be applied to obtain either (1) lipid class concentrations, if AIF is used and samples are quantified with ESTDs (only Splash at different dilution levels), or (2) lipid species concentration if samples are measured in MS1 with Splash as ISTD. In both methods, 5 µL per sample was injected and source parameters were kept the same: spray voltage 3.5 kV (pos)/ -2.8 kV (neg), capillary temperature 280°C, sheath gas flow rate of 38, auxiliary gas flow rate of 3, sweep gas was switched off, auxiliary gas heater temperature of 320°C and S-lens radio frequency level of 30. In MS1 mode the mass range in both polarities was set to *m/z* 400-1000 and in AIF to *m/z* 100-1000. Both had a maximum injection time (IT) of 200 ms, a resolution of 120000, and an automatic gain control (AGC) target of 3e6. In AIF, the normalized collision energy (NCE) was set to 25 (pos)/ 28 (neg). Skyline (version 20.2) was used for MS1 data processing and AIF fatty acyl chain fragments whereas head fragments in the AIF files were integrated manually with Qual Browser Thermo Xcalibur™ (version 4.0.27.19).

Concentrations of the lipid species in MS1 mode were calculated as in formula 2.

(2) $$conc\ \left[\frac{\mu mol}{1\ mL \approx 1.6\ 10^{10}\ cells}\right] = \frac{Area_{\ Analyte}}{Area_{ISTD}} \cdot conc_{ISTD}\left[{}^{\mu mol}/_{mL}\right] \cdot Dilution\ Factor$$

Depending on the lipid class, different fragmentation led to different possibilities summarized in Supplementary Table 1 and exemplarily shown in Figure 1 (manuscript). A headgroup fragment is either present as a product ion (e.g. m/z 184 for PC) or neutral loss (e.g. m/z 141 for PE). For neutral losses, different fatty acyl chain compositions lead to different product ions and a mass range for

integration is necessary. In negative mode, the FAs present in yeast were selectively integrated with Skyline, and the sum area was used for quantification.

Sample concentrations were calculated via linear regression models of ESTDs and the results were multiplied with the dilution factor (1:1000). LOD was calculated by multiplying the standard deviation of 3 repetitive injections of a low concentrated Splash ESTD with 3.

For lipid species quantification in MS1 mode, identified lipids were removed if the absolute mass error was above 3 and if the concentration was below LOQ. LOQ values were calculated by multiplying the standard deviation of 5 repetitive injections of a low concentrated Splash ISTD with 10.

Shotgun analysis (lipid species quantification with ISTD)

Shotgun analysis was conducted as previously described at Schoeny *et al.*[2] Briefly, a high field Q Exactive HF™ quadrupole-Orbitrap mass spectrometer (Thermo Fisher Scientific) was connected with a robotic nanoflow ion source TriVersa NanoMate® (Advion BioSciences, Ithaca NY, USA) and a nanoelectrospray ionization (nanoESI) chip with spraying nozzles of 5 µm nominal internal diameter. IPA/MeOH/$CHCl_3$ (4:2:1, v/v/v) containing 7.5 mM ammonium formate was used for sample dissolving and dilution. SPLASH® Lipidomix® ISTD was added in a 1:500 dilution to a 1:1000 diluted yeast sample and a final volume of 200µL. Three replicates of 30 µL were placed in a 96 twin.tec® well plate (Eppendorf, Hamburg, Germany). Following settings were applied in the Chipsoft 8.3.1 software (Advion BioSciences): ionization voltage 1.25 kV (pos)/ -1.25 kV (neg); backpressure 0.9 psi and in the MS source parameters: capillary temperature 250 °C, S-Lens radio frequency level 50. A 9 min polarity switching method with data independent acquisition (DIA) and MS1 scans was used with the following settings: resolution 240,000 (MS1), 60,000 (MS2), AGC target 1e6 (MS1), 2e5 (MS2), maximum IT 150 ms (MS1), 130 (MS2), scan range in MS1 *m/z* 350-1050 (pos)/ *m/z* 200-1200 (neg), NCE for MS2 of 21 (pos)/ 26 (neg) and a fixed first mass of *m/z* 80 (pos)/ *m/z* 150 (neg). LipidXplorer 1.2.8 settings were the following: mass tolerance 5 ppm, min. occupation of 0, intensity threshold 10000 (MS1)/ 5000 (MS2), resolution 260000 (MS1)/ 65000 (MS2), resolution gradient -102 (MS1)/ -60 (MS2). Identified lipids were removed if the absolute mass error was above 3, if isobaric compounds were present and if the concentration was below LOQ. LOQ values were calculated by multiplying the standard deviation of 5 repetitive injections of a low concentrated Splash ISTD with 10.

RP-LC-MS analysis (lipid species quantification with ISTD)

A Vanquish™ Horizon HPLC (Thermo Fisher Scientific) with an Acquity HSS T3 (2.1 mm × 150 mm, 1.8 µm, Waters, Milford, MA, USA) with a VanGuard Pre-column (2.1 mm × 5 mm, 100 Å, 1.8 µm) was used for reversed-phase liquid chromatography-high resolution mass spectrometry (RP-LC-HRMS). The flow rate was set to 250 µL min^{-1} and the column temperature to 40°C. Solvent A was ACN/H_2O (3:2, v/v), and solvent B was IPA/ACN (9:1, v/v). Both solvents contained 0.1% formic acid and 10 mM ammonium formate. The following gradient was used: 0−2 min 30% B, 2−15 min ramp to 75% B, 15−17 min ramp to 100% B, 17−22 min 100% B and 22-27 min 30% B as equilibration step. The injection volume was 5 µL and the injector needle was washed with 75% IPA, 24.9% H_2O, and 0.1% formic acid before each injection.

A high field Q Exactive HF™ quadrupole-Orbitrap mass spectrometer (Thermo Fisher Scientific) was used as MS. The following source parameters were applied: capillary temperature of 220°C (pos)/ 250°C (neg), sheath gas flow rate of 48 (pos)/ 35 (neg), an auxiliary flow rate of 10, sweep gas of 2, S-lens RF level of 40 and auxiliary gas heater temperature of 300 °C applying a spray voltage of 3.5 kV in positive mode and 2.8 kV in negative mode. In MS1 mode the mass range in both polarities was set to *m/z* 200-2000. A maximum IT of 200 ms, a resolution of 120000, and an AGC target of 1e6 was applied. Skyline (version 20.2) was used for peak integration and R/ R studio for final data processing.

Identified lipids were removed if the absolute mass error was above 3 and if the concentration was below LOQ. LOQ values were calculated by multiplying the standard deviation of 5 repetitive injections of a low concentrated Splash ISTD with 10.

Bibliography

(1) Neubauer, S.; Haberhauer-Troyer, C.; Klavins, K.; Russmayer, H.; Steiger, M. G.; Gasser, B.; Sauer, M.; Mattanovich, D.; Hann, S.; Koellensperger, G. U13C Cell Extract of Pichia Pastoris - A Powerful Tool for Evaluation of Sample Preparation in Metabolomics. *J. Sep. Sci.* **2012**, *35* (22), 3091–3105.

(2) Schoeny, H.; Rampler, E.; Hermann, G.; Grienke, U.; Rollinger, J. M.; Koellensperger, G. Preparative Supercritical Fluid Chromatography for Lipid Class Fractionation — a Novel Strategy in High-Resolution Mass Spectrometry Based Lipidomics. *Anal. Bioanal. Chem.* **2020**, *412*, 2365–2374.

(3) Kato, T.; Nishimiya, M.; Kawata, A.; Kishida, K.; Suzuri, K.; Saito, M.; Fujita, K.; Igarashi, T.; Inagaki, M. Quantitative 31P NMR Method for Individual and Concomitant Determination of Phospholipid Classes in Polar Lipid Samples. *J. Oleo Sci.* **2018**, *67* (10), 1279–1289.

TABLES

Table 1 Overview of detected lipid classes in HILIC-AIF. LOD was calculated by multiplying the standard deviation of a low standard by 3, LLOQ and ULOQ correspond to the borders of the linear range (the lowest and the highest standard in this range). *indicate lipids with double peaks, the RT of the highest is shown.

Lipid class	Type	Fragment	Formula	Mass (range)	RT [min]	[µmol L^{-1}] LOD	LLOQ	ULOQ	R^2
PC	HG	$[HG]^+$	$[C_5H_{15}O_4PN]^+$	184.0733	4.04	0.017	0.2	4	0.9973
LPC	HG	$[HG]^+$	$[C_5H_{15}O_4PN]^+$	184.0733	7.48*	0.010	0.05	9	0.998
SM	HG	$[HG]^+$	$[C_5H_{15}O_4PN]^+$	184.0733	6.58	-	0.04	8	0.9972
PI	HG	$[HG]^-$	$[C_6H_{11}O_8P]^-$	241.0119	8.86	0.133	0.1	2	0.9956
PE	HG	$[M-HG]^+$	$[M-C_2H_6O_4PN]^+$	570-605	6.0	0.001	0.007	7	0.9996
PG	HG	$[M-HG]^+$	$[M-C_2H_6O_4PN]^+$	570-605	2.9	0.003	0.035	0.7	0.9981
PC	FAs	$[FA+O]^-$	$[C_XH_YO_2]^-$	241-283	4.04	0.082	0.2	4	0.9971
LPC	FAs	$[FA+O]^-$	$[C_XH_YO_2]^-$	241-283	7.48*	0.011	0.045	9	0.9996
LPE	FAs	$[FA+O]^-$	$[C_XH_YO_2]^-$	241-283	9.47*	0.112	0.04	20.1	0.999
PE	FAs	$[FA+O]^-$	$[C_XH_YO_2]^-$	241-283	6.0	-	0.007	1.5	0.9993
PG	FAs	$[FA+O]^-$	$[C_XH_YO_2]^-$	241-283	2.9	0.013	0.03	0.7	0.9956

Table 2 lipid class concentration of *K. phaffii.* Data obtained by lipid class quantification methods (^{31}P-NMR, HILIC-ICP-MS, HILIC-ESI-AIF headgroup, and FA) only. Uncertainty is shown for classes quantified with more than one method.

Lipid class	Nr. Methods	Conc. [µmol/ 1.6 10^{10}cells]
**PC**	4	1218±17
**PE**	4	594±8
**PS**	1	293
**PI**	1	119
**LPC**	2	21±2.3
**PG**	3	18±1.6

Table 3 Absolute concentration values of different lipid classes and their overall value across all platforms.

Method [µmol/ 1.6 10^{10}cells]	PC	PE	PI	PS	PG	LPC
^{31}P-NMR	1227±34	702±124	119±33	293±74	-	-
HILIC-ICP-MS	1261±39	637±26	-	-	21±0.8	-
HILIC-ESI-AIF-FAs	1222±30	616±16	-	-	27±5	23±5
HILIC-ESI-AIF-Head	1157±38	580±9	-	-	16±0.5	21±3
RP-LC-ESI-MS	1291±31	663±15	94±4	301±14	27±0.7	29±0.7
Shotgun	1231±206	603±121	100±32	269±38	21±8	21±0.5
HILIC-ESI-MS1	1235±30	479±40	-	-	19±1.5	23±1.3
Mean± uncertainty (CoV)	1234±14 (1.1%)	605±6 (1.0%)	94±2 (2.1%)	300±13 (4.3%)	21±2 (9.5%)	23.5±0.4 (1.7%)

FIGURES

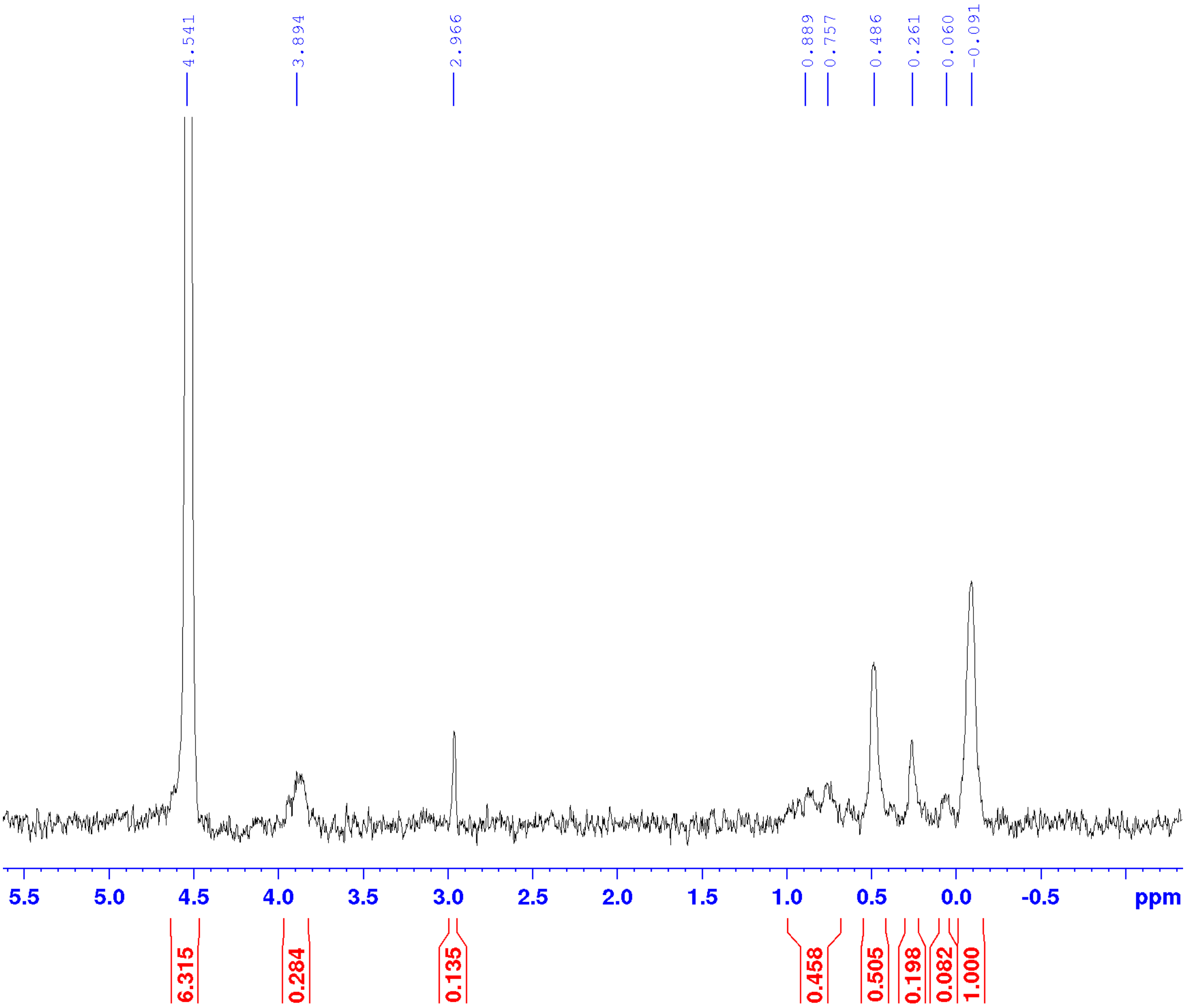

Figure 1 ^{31}P-NMR spectrum of 1.6 10^{10}yeast cells in 2 mL solution. Peak identification: PC -0.091; PI 0.06; PS 0.261; PE+LPC 0.486; PG+LPE 0.757+0.889; phosphoserine -ISTD 4.541; Impurity 2.966; PA 3.894; noise value was selected at 5.793-5.291.

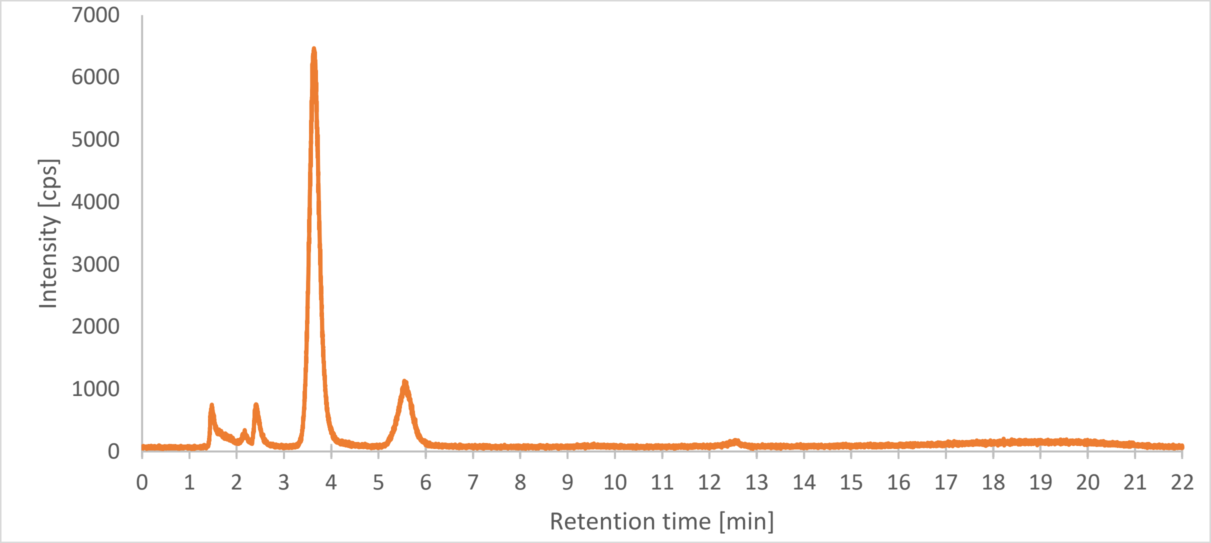

Figure 2 HILIC-ICP-MS chromatogram of yeast extract. Peak identification: Void volume 1.6 min; PG 2.5 min; PC 3.7 min; PE+PA 5.6 min.

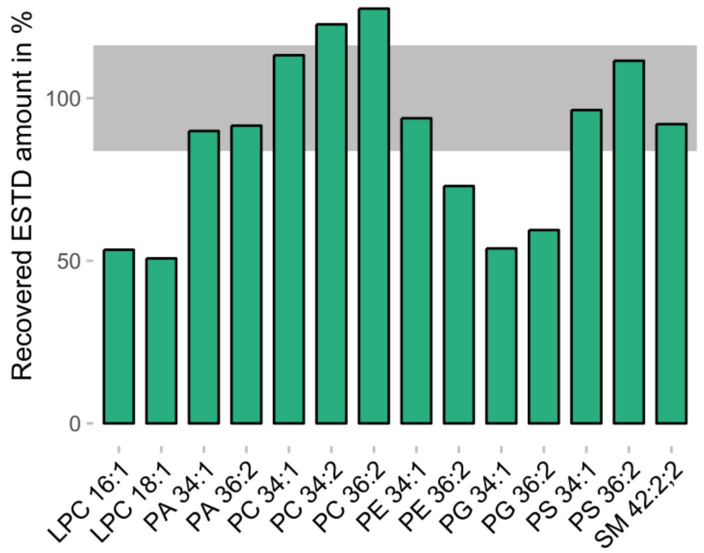

Figure 3 Recovery of two-year-old ESTDs analyzed via FI-ICP-MS and external calibration. ESTD had been stored at -80°C in chloroform/methanol solution. Grey area corresponds to ±9% precision via ICP measurements. Lower values indicate degradation, higher values can be explained by solvent evaporation.

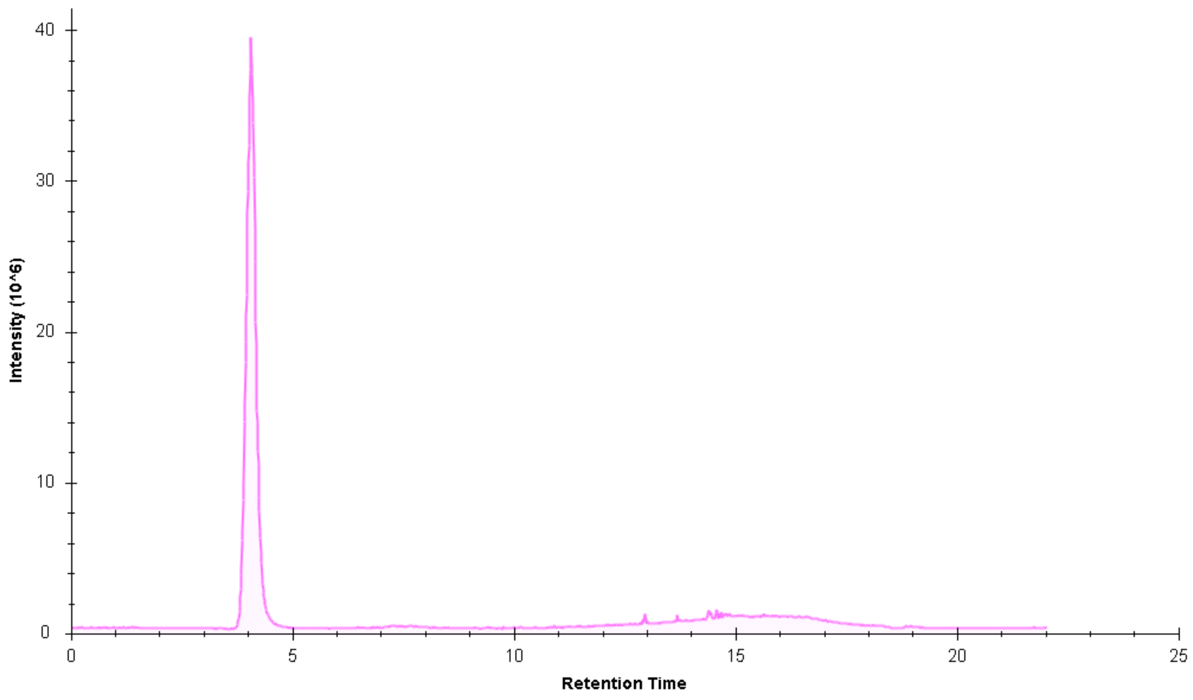

Figure 4 HILIC-ESI-AIF chromatogram of yeast extract in positive mode showing the mass trace of m/z 184.

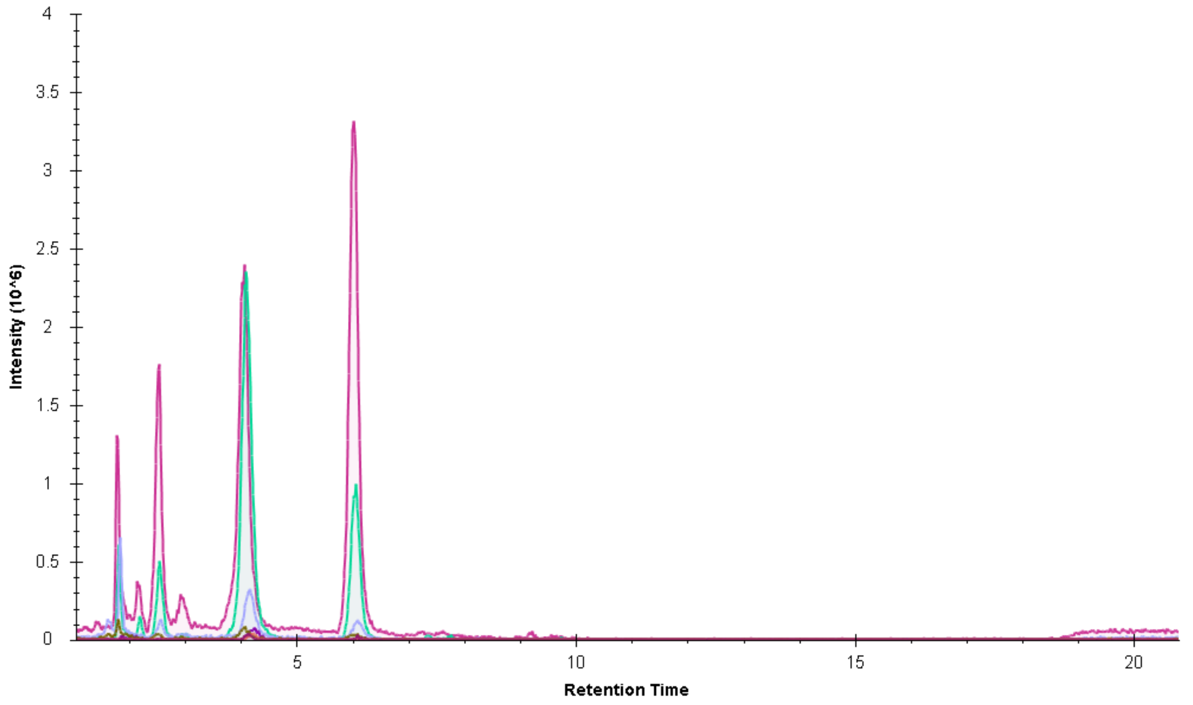

Figure 5 HILIC-ESI-AIF chromatogram of yeast extract in negative mode showing the mass traces of the different fatty acyl chain fragments.

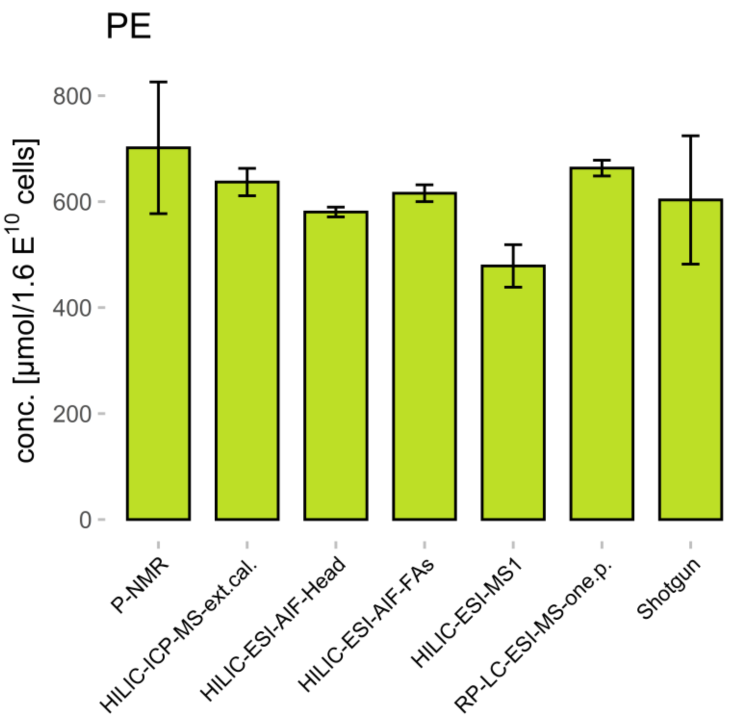

Figure 6 Absolute concentration values of PE in µmol/ 1.6 10^{10} yeast cells (equals 1 g wet weight of yeast cells). Error bars indicate technical repeatability (n=3).

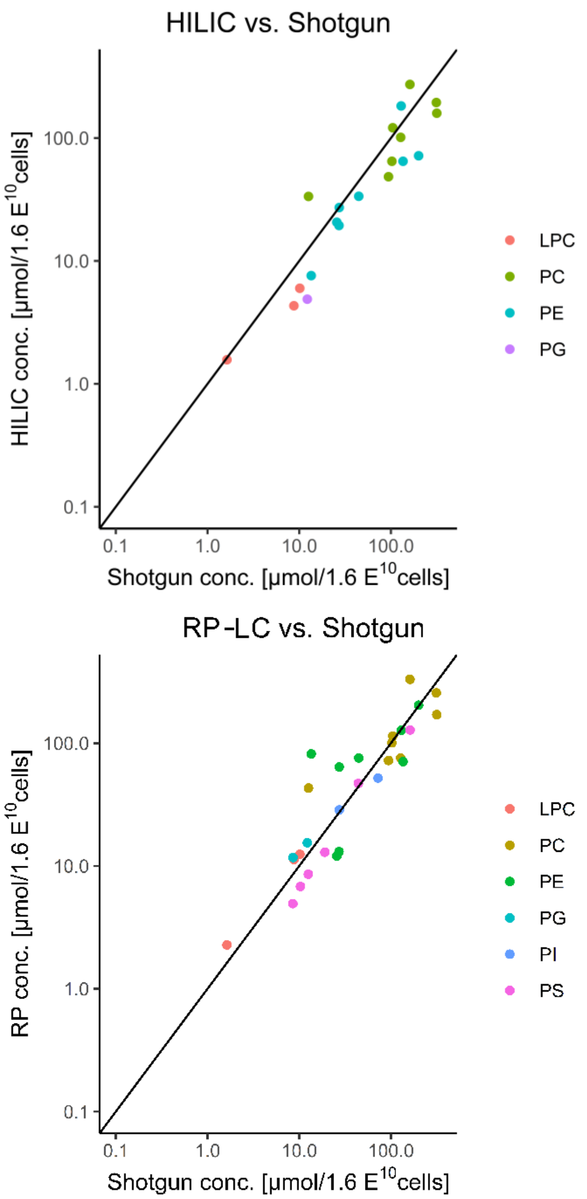

Figure 7 Correlation plot between the absolute concentrations obtained from the three lipid species quantification methods. Quantified yeast lipids are shown if present in the two compared methods. The line indicates y=x. Colors indicate the lipid class of the different analytes.
